# PReS-FINAL-2047: Exploring the effectiveness of stretch and cast treatment of fixed flexion deformity in children with active JIA

**DOI:** 10.1186/1546-0096-11-S2-P60

**Published:** 2013-12-05

**Authors:** E Haggart, S Maillard

**Affiliations:** 1 Rheumatology Physiotherapy, Great Ormond Street Hospital NHS Foundation Trust, London, UK

## Introduction

Active JIA of the knee can lead to bony overgrowth and leg length discrepancies in children with JIA. Fixed flexion deformities of the knee result in loss of inner range quadriceps strength, abnormal gait and reduced independent function and participation in sports and activities.

## Objectives

Our objective was to review the use of stretch and cast procedures alongside Intra-articular steroid injections (IAI) for active JIA of the knee resulting in fixed flexion deformities (FFD) of greater than 15 degrees.

## Methods

A retrospective review was undertaken of all patients presenting to Great Ormond Street Hospital NHS Foundation Trust between 2005-2013 with active JIA of the knee resulting in FFD and loss of knee extension range of movement (ROM) greater than -15 degrees extension and underwent a stretch and cast procedure alongside IAI for treatment of their disease. ROM was assessed pre and post procedure. Patients received their IAI under general anaesthetic, and then the physiotherapists carried out a stretch and cast procedure.

The stretch and cast procedure included patella mobilisations, gentle and prolonged knee extension passive stretching, and casting with a fibre-glass cast for 72 hours. All patients then received 2 weeks of intensive physiotherapy strengthening. ROM and inner range quadriceps strength were measured at the end of these 2 weeks post procedure.

## Results

30 patients (9 male, 21 female; 15 Oligo JIA, 10 Poly JIA, 3 EOJIA) had a FFD of between -15 and -90 degrees (mean -30) extension at the affected knee(s). A total of 35 joints were injected and underwent the stretch and cast procedure plus physiotherapy strengthening. Post intensive rehab the patients all had improved or normal ROM (mean -7 degrees) and improved muscle strength (mean IRQ 8/10 Kendall Scale) in order to maintain this improvement. This was maintained at 4-6 month follow up in 94% of patients. The 2 patients who did not maintain range of movement had poor compliance to their physiotherapy home exercise programme.

## Conclusion

ROM improved following procedure in all patients, indicating that stretch and cast treatment is an effective intervention for the management of fixed flexion deformities secondary to active JIA. Ongoing strengthening of inner range quadriceps is vital to maintain this improved range of movement and restoration of function and normal gait.

## Disclosure of interest

None declared.

